# Editorial: Biodiversity and conservation of fungi and fungus-like organisms

**DOI:** 10.3389/ffunb.2022.973249

**Published:** 2022-12-14

**Authors:** Danny Haelewaters, Yusufjon Gafforov, Li-Wei Zhou

**Affiliations:** ^1^ Research Group Mycology, Department of Biology, Ghent University, Ghent, Belgium; ^2^ Faculty of Science, University of South Bohemia, České Budějovice, Czechia; ^3^ Department of Ecology and Evolutionary Biology, University of Colorado Boulder, Boulder, CO, United States; ^4^ Laboratory of Mycology, Institute of Botany, Academy of Sciences of Republic of Uzbekistan, Tashkent, Uzbekistan; ^5^ AKFA University, Tashkent, Uzbekistan; ^6^ State Key Laboratory of Mycology, Institute of Microbiology, Chinese Academy of Sciences, Beijing, China

**Keywords:** dark taxa, funga, fungal conservation, fungal diversity, integrative taxonomy approach, phenology

The second species-richest group after insects, fungi and fungus-like organisms, play essential roles as saprotrophs, mutualists, and parasites and pathogens. Fungi thrive in almost every ecosystem on earth; the total number of fungal species has been estimated at 1.5 to 6 million (Hawksworth, 1991; Taylor et al., 2014; Hawksworth and Lücking, 2017). However, only 148,000 species, less than 10% of the estimated diversity, are formally described (Hibbett et al., 2016; Cheek et al., 2020). In addition, knowledge is incomplete even for the named species, as their geographic distributions and host ranges are often not fully understood. Finally, phylogenetic relationships among fungi, especially early diverging lineages, lack support and are continuously amended (Naranjo-Ortiz and Gabaldón, 2019; Wijayawardene et al., 2020). These knowledge gaps limit the utilization of fungal resources and the development of pathogenic and parasitic fungi as part of integrated biological control strategies. They also hamper conservation efforts (Gonçalves et al., 2021; Bazzicalupo et al., 2022); if we do not know what is present, how can we protect it?

There is a need to accelerate the description of fungi and fungus-like organisms and to facilitate the conservation of fungi and utilization of their resources. These organisms should be studied and documented with the help of standardized methods, including field monitoring, macro- and micromorphological observations, and molecular phylogenetic reconstructions. In addition, when available, documentation may incorporate data from culture studies and high-throughput sequencing techniques. The integrative (or polyphasic) taxonomy approach – incorporating all available, independent lines of evidence – will further benefit the field in addressing broader questions relating to host specificity patterns, biological control, ecology, and fungal systematics (Cao et al., 2021; Maharachchikumbura et al., 2021). As the mycological community will move forward describing fungal diversity based on recommended best practices (Aime et al., 2021), the Fungal Tree of Life should continue to be populated by taxa with multilocus phylogenetic data and, where available, phylogenomic data (McLaughlin et al., 2009; Spatafora et al., 2017; James et al., 2020).

Each paper in this Research Topic is closely tied to promoting fungal biodiversity and conservation. In their perspective, Cazabonne et al. call for integrating field inventory studies and molecular methods (also see Truong et al., 2017). The authors point out the importance of field-based research for taxonomy, documentation of new geographic records, ecology, culturing approaches, fungarium collections, checklists, and training of junior mycologists as well as parataxonomists. Stallman and Robinson focus on phenological patterns of Hawaiian mushrooms and, based on their results, suggest adding seasonality data to IUCN Red List assessments.

Some taxonomic and functional groups of fungi have traditionally been neglected. Examples are the arthropod-associated Laboulbeniomycetes (Haelewaters et al., 2022), early-diverging fungi (Spatafora et al., 2016), and fungal hyperparasites. Van Caenegem et al. study Laboulbeniales microfungi associated with ladybirds using morphology, single-locus and multilocus phylogeny, and host association. The authors show that segregation of species within the *Hesperomyces virescens* species complex is governed not only by host but also by a geographic component. Bermúdez-Cova et al. present a checklist of hyperparasitic fungi in association with black mildews (Meliolales, Sordariomycetes). Their review reveals an assemblage of seven morphological groups that are poorly represented in public sequence databases. Finally, Blaalid and Davey focus on overlooked fungal diversity of protected coastal heathland sites in Norway. The authors discuss incorporating fungi into holistic conservation strategies based on well-developed protocols, involving standardized monitoring and carefully designed sampling strategies to provide reliable biodiversity estimates.

## Author contributions

DH: writing—original draft and visualization. DH, YG, and L-WZ: writing—review and editing. All authors contributed to the article and approved the submitted version.

## Funding

We received support from the Research Foundation–Flanders (Junior Postdoctoral Fellowship 1206620N to DH), the State Scientific and Technical Program of the Republic of Uzbekistan (2021–2024, YG), and the Ministry of Innovative Development of the Republic of Uzbekistan (project no. AL 2021090820 to YG).

## Acknowledgments

We thank the 15 authors and 12 reviewers and editors who contributed to this Research Topic, as well as the staff at both Frontiers journals for administrative and editorial support.

## Conflict of interest

The authors declare that the work was conducted in the absence of any commercial or financial relationships that could be construed as a potential conflict of interest.

## Publisher’s note

All claims expressed in this article are solely those of the authors and do not necessarily represent those of their affiliated organizations, or those of the publisher, the editors and the reviewers. Any product that may be evaluated in this article, or claim that may be made by its manufacturer, is not guaranteed or endorsed by the publisher.
